# Genetic correlates of brain aging on MRI and cognitive test measures: a genome-wide association and linkage analysis in the Framingham study

**DOI:** 10.1186/1471-2350-8-S1-S15

**Published:** 2007-09-19

**Authors:** Sudha Seshadri, Anita L DeStefano, Rhoda Au, Joseph M Massaro, Alexa S Beiser, Margaret Kelly-Hayes, Carlos S Kase, Ralph B D'Agostino, Charles DeCarli, Larry D Atwood, Philip A Wolf

**Affiliations:** 1 The National Heart Lung and Blood Institute's Framingham Heart Study, Framingham, MA, USA; 2 Department of Neurology, Boston University School of Medicine, Boston, MA, USA; 3 Department of Biostatistics, Boston University School of Public Health, Boston, MA, USA; 4 Statistics and Consulting Unit, Department of Mathematics, Boston University, Boston, MA, USA; 5 The Department of Neurology, University of California – Davis, Sacramento, CA, USA

## Abstract

**Background:**

Brain magnetic resonance imaging (MRI) and cognitive tests can identify heritable endophenotypes associated with an increased risk of developing stroke, dementia and Alzheimer's disease (AD). We conducted a genome-wide association (GWA) and linkage analysis exploring the genetic basis of these endophenotypes in a community-based sample.

**Methods:**

A total of 705 stroke- and dementia-free Framingham participants (age 62 +9 yrs, 50% male) who underwent volumetric brain MRI and cognitive testing (1999–2002) were genotyped. We used linear models adjusting for first degree relationships via generalized estimating equations (GEE) and family based association tests (FBAT) in additive models to relate qualifying single nucleotide polymorphisms (SNPs, 70,987 autosomal on Affymetrix 100K Human Gene Chip with minor allele frequency ≥ 0.10, genotypic call rate ≥ 0.80, and Hardy-Weinberg equilibrium p-value ≥ 0.001) to multivariable-adjusted residuals of 9 MRI measures including total cerebral brain (TCBV), lobar, ventricular and white matter hyperintensity (WMH) volumes, and 6 cognitive factors/tests assessing verbal and visuospatial memory, visual scanning and motor speed, reading, abstract reasoning and naming. We determined multipoint identity-by-descent utilizing 10,592 informative SNPs and 613 short tandem repeats and used variance component analyses to compute LOD scores.

**Results:**

The strongest gene-phenotype association in FBAT analyses was between *SORL1 *(rs1131497; p = 3.2 × 10^-6^) and abstract reasoning, and in GEE analyses between *CDH4 *(rs1970546; p = 3.7 × 10^-8^) and TCBV. *SORL1 *plays a role in amyloid precursor protein processing and has been associated with the risk of AD. Among the 50 strongest associations (25 each by GEE and FBAT) were other biologically interesting genes. Polymorphisms within 28 of 163 candidate genes for stroke, AD and memory impairment were associated with the endophenotypes studied at p < 0.001. We confirmed our previously reported linkage of WMH on chromosome 4 and describe linkage of reading performance to a marker on chromosome 18 (GATA11A06), previously linked to dyslexia (LOD scores = 2.2 and 5.1).

**Conclusion:**

Our results suggest that genes associated with clinical neurological disease also have detectable effects on subclinical phenotypes. These hypothesis generating data illustrate the use of an unbiased approach to discover novel pathways that may be involved in brain aging, and could be used to replicate observations made in other studies.

## Background

Age-related neurological diseases such as stroke and dementia represent a substantial population burden, and one in three persons will develop either stroke or dementia in their lifetime [1]. Twin studies suggest that 37–78% of the variance in the age of onset of Alzheimer's disease (AD), the most common cause of dementia in the elderly, can be attributed to additive genetic effects [2,3]. Conversely, cognitively healthy aging also has a substantial genetic basis [4]. Finally ischemic stroke [5-7] and vascular cognitive impairment are also heritable [8]. However, surprisingly few genes have been identified that determine the risk of developing stroke (*PDE4D*, *ALOX5AP*) [9-11] or Alzheimer's disease (*APOE4*) [12], in the community as a whole, that is for persons not from autosomal dominant, early-onset families. One reason may be that studies to date have been underpowered to detect small effects. Two additional challenges to a more complete understanding of the genetic basis of these aging related brain diseases have been the late phenotypic manifestation of these conditions and their complex, polygenic mode of inheritance. Multiple genes interacting with each other and with environmental factors likely create a complex gradient of susceptibility to disease. We hypothesized that studying the genetic basis for the gradient of susceptibility underlying AD and stroke, using endophenotypes, would provide insights into the genetics of these late-onset neurological diseases. Endophenotypes (or intermediate phenotypes) are heritable traits that reveal the actions of genes predisposing an individual to develop a disease but they often manifest years before clinical and pathological diagnostic criteria for the disease are met.

Volumetric brain MRI and comprehensive cognitive testing have been used to define heritable, reproducible, quantitative endophenotypes which in turn relate to the risk of developing dementia or stroke [13-19]. Twin studies have demonstrated substantial heritability of these endophenotypes [20]. The recent availability of high-throughput platforms permits genome-wide association studies (GWAS) that incorporate a more comprehensive and unbiased approach to detect genes with modest phenotypic effects. We present the results of a GWAS of structural and functional phenotypes previously associated with cellular and vascular brain aging.

## Methods

### Study sample

The study design, selection criteria and participant demographics of the Framingham Original and Offspring cohorts have been detailed in prior publications [21,22]. A total of 1345 persons, who were members of the 330 largest families across these two cohorts, underwent genotyping using the Affymetrix GeneChip Human Mapping 100K single nucleotide polymorphism (SNP) set. The Overview provides details of this sample [23]. The study sample for the current analyses comprised of 705 stroke- and dementia-free Framingham Study participants who were genotyped and had undergone volumetric brain MRI and/or cognitive testing between 1999 and 2002. Among the 1345 eligible persons who were genotyped, 508 persons were excluded since they died prior to their 7^th ^Offspring examination, did not attend this examination, declined or were unable to complete MRI or cognitive testing, 12 persons were excluded for prevalent stroke (n = 12) at the time of MRI and cognitive testing and 11 persons with neurological diseases such as multiple sclerosis or brain tumor that could impact study phenotypes were also excluded; all participants were screened, but none required exclusion for dementia at the time of MRI. Nine individuals were excluded because covariate information was not available. This study was approved by the Institutional Review Board of Boston University Medical Center; all participants provided written informed consent including consent for genetic studies.

### Phenotype definition

The list of study phenotypes is shown in Column 1 of Table 1.

**Table 1:** Structural (Volumetric MRI) and Functional (Cognitive Testing) Brain Aging Phenotypes

			Exam cycle		
**Phenotypic Trait (Abbreviated Variable Name)**	**Number of traits**	**N**	**Original Cohort (Exam 26)**	**Offspring Cohort (Exam 7)**	**Covariates Used to Create Multivariable-Adjusted Residuals**

Volumetric Brain MRI*					

Total Cerebral Brain Volume (ATCBV)	1	705	Original Cohort & Offspring data are pooled		Age, age-squared, sex, current smoking status, diabetes, systolic blood pressure, anti-hypertensive drugs, atrial fibrillation, EKG-LVH at Offspring examination 7 and Original cohort 26; data from sex-specific regressions pooled.
Frontal Brain volume (AFBV)	1	705			
Parietal Brain Volume (APBV)	1	705			
Occipital Brain Volume (AOBV)	1	705			
Temporal Brain volume (ATBV)	1	705			
Hippocampal Volume (AHPV)	1	327			
Lateral Ventricular Volume (ALVV)†	1	705			
Temporal Horn Volume (LTHBV)†	1	705			
White Matter Hyperintensity Volume (BMRIZLWMHVMV) [Age-, sex-specific Z-score of log-normalized white matter hyperintensity volume]	1	705			Current smoking status, diabetes, systolic blood pressure, anti-hypertensive drugs, atrial fibrillation, EKG-LVH at Offspring examination 7 and Original cohort 26; data from sex-specific regressions pooled.

**Cognitive Test Performance**					

Factor 1:Verbal Memory (F1)	1	694	Original cohort and Offspring data are pooled		Birth cohort by decade, education, Framingham Stroke Risk Profile score, plasma homocysteine concentrations (at the 20^th ^Original cohort and the 6^th ^Offspring examinations), apolipoprotein E genotype (ε4 +ve/-ve); data from sex-specific regressions were pooled.
Factor 2:Visual Memory and Organization (F2)	1	694			
Factor 3: Measure of attention and executive function-Trails A and B (F3)	1	694			
Boston Naming Test (Nam)	1	694			
Similarities (Sim)	1	694			
Wide-Range Achievement Test (WRAT)	1	694			

*All MRI volumes were expressed as a ratio of total intracranial volume (TCV), trait names used in this table correspond to trait names posted at the website; an 'A' preceding the trait name refers to the multivariable adjusted residual.

† Log-normalized values of these traits were used. Website http://www.ncbi.nlm.nih.gov/projects/gap/cgi-bin/study.cgi?id=phs000007.

### Volumetric brain MRI

Details of brain MRI acquisition parameters, blinded image analysis, definition of brain volumes (indexed for cranial cavity size) and the mean and standard deviation (SD) values for these measures in the larger sample of all Framingham subjects (n = 2259) who underwent brain MRI, have been published previously [14,15,24-27]. Mean and SD values and heritability estimates for each of these parameters in the current study sample are available online at http://www.ncbi.nlm.nih.gov/projects/gap/cgi-bin/study.cgi?id=phs000007. Digital information from the MRI scans was transferred to a central laboratory directed by one of the authors (C.D.) for processing and analysis. Analysis was done blind to the subjects' genotype, demographic and vascular risk factor data. Analyses were done using semi-automated measurements of pixel distributions based on mathematical modeling of MRI pixel intensity histograms for cerebrospinal fluid and brain matter (white matter and gray matter) to determine the optimal pixel intensity threshold that distinguished cerebrospinal fluid (CSF) from brain matter. Brain volume was determined in coronal sections by manually outlining the intracranial vault above the tentorium to determine the total cranial volume (TCV). Next, the skull and other non-brain tissues were removed from the image, followed by mathematical modeling to determine total brain volume (TBV). TBV included the supratentorial gray and white matter and excluded the CSF. We used the ratio of TBV to TCV (Total Cerebral Brain Volume, TCBV) as a measure of brain volume to correct for differences in head size. Regional brain volumes were measured as the sum of the segmented right and left lobar volumes for that region indexed to the intracranial volume; frontal (FBV), parietal (PBV), occipital (OBV) and temporal (TBV) lobar brain volumes and the regional brain volume of the hippocampus (based on hand-drawn outlines) were assessed. Two measures of ventricular volume were used: the lateral ventricular volume, and the temporal horn volume each of which was measured as the sum of the volumes for two sides, log-normalized and indexed over TCV. Finally the white matter hyperintensity volume was measured as a z-score within 10-year age- and sex-specific categories of the logarithmically transformed continuous variable (WMH). All analyses were performed using a custom-designed image analysis package, QUANTA 6.2, operating on a Sun Microsystems (Santa Clara, CA) Ultra 5 workstation. The inter-rater reliabilities ranged between 0.90 and 0.94 for TCV, TCB, regional brain and ventricular volumes and white matter hyperintensities, and intra-rater reliabilities average 0.98 across all measures.

### Cognitive measures

Subjects were administered a neuropsychological test battery using standard administration protocols and trained examiners. Details of the tests administered and normative values for the Framingham Original and Offspring cohorts have been previously published [13,28]. Since individual cognitive tests are scored measured on different scales and since scores are known to be associated with age and sex, we transformed the variables, separately by sex, to obtain variables that are comparable across tests. First, natural logarithmic transformations were applied to normalize raw scores that had a skewed distribution. Next, each variable was regressed on age and residuals from these regressions were standardized using a z-score transformation. The resulting standardized cognitive test scores were then either summed to create 3 factors, each characterizing a specific cognitive domain: verbal memory (Factor 1, F1), visuospatial memory and organization (Factor 2, F2) and attention and executive function (Factor 3, F3), or were used individually (Similarities [Sim], Boston Naming Test [BNT] and Wide Range Achievement Tests [WRAT]). Details of test source and parameters used to define each individual test and factor are outlined in Additional data file 1, table 1.

### Genotyping

The Overview [23] describes the Affymetrix 100K SNP GeneChip genotyping http://gmed.bu.edu/about/genotyping.html and the Marshfield short-tandem repeat genotyping performed by the Mammalian Genotyping Service http://research.marshfieldclinic.org/genetics. Only the SNP data were used for GWA studies whereas both SNP and STR data were combined for linkage analyses.

### Statistical analysis

As detailed in the Overview [23], we used linear models adjusting for first degree relationships via generalized estimating equations (GEE) and family based association tests (FBAT). All tests were performed using additive genetic models to relate qualifying SNPs to multivariable-adjusted residuals of the 9 MRI measures and the 6 cognitive factors/tests described earlier. Qualifying SNPs (n = 70,897) were defined as autosomal SNPs with genotypic call rate ≥80%, minor allele frequency ≥10% and in Hardy-Weinberg equilibrium with p ≥ 0.001. Additionally, for FBAT analyses ≥10 informative families were required. For the linkage analyses, we used Merlin software to compute multipoint identity-by-descent utilizing 10,592 informative SNPs and 613 short tandem repeats selected to minimize LD [29,30]; we then used maximum variance component analyses in SOLAR to compute LOD scores as a measure of linkage [31].

Multivariable-adjusted trait residuals for the phenotypic traits listed in Table 1 were computed using linear regression and the full set of all Framingham Study participants in whom the phenotype of interest was available. For the MRI analyses, residuals were derived from multivariable linear regressions in SAS [32], adjusting for the variables that we had previously found were related to MRI measures: age and if appropriate age-squared, current smoking status, systolic blood pressure in mm Hg, use of anti-hypertensive drugs and presence or absence of diabetes mellitus, atrial fibrillation and electrocardiographic left ventricular hypertrophy. Similarly, residuals were derived for each cognitive measure from multiple linear regressions and adjusting for the following covariates: birth cohort by decade, education (high school, high school graduate, some college or college graduate), Framingham Stroke Risk Profile score, plasma homocysteine concentrations (at the 20^th ^Original cohort and the 6^th ^Offspring examinations) and apolipoprotein E genotype (ε4 +ve/-ve). Unless otherwise specified, covariate data for all 15 phenotypic measures were drawn from the 26^th ^Original cohort and the 7^th ^Offspring examinations. Data from sex-specific regressions were pooled for the SNP-phenotype association and linkage analyses. Winsorized residuals (truncating extreme values at ± 3.5 standard deviations) were used for linkage analysis of phenotypes with departures from normality as assessed by skewness and kurtosis (TBV, temporal horn volume, F1, F2, F3, Sim, BNT and WRAT).

### Presentation of results

We used several strategies to explore the resulting phenotype-SNP association and linkage results. First, we used an unbiased approach and collated the 50 strongest phenotype-SNP associations (those with the smallest p-value) including 25 phenotype-SNP associations each for GEE and FBAT analyses, and all linkage results with a LOD score > 2.0. All SNPs were annotated using the UCSC genome browser tables http://genome.ucsc.edu/ [33,34] to examine if the SNP was within a gene and to identify this gene.

Next, we examined the data for genes with pleiotropic effects. We assessed if genes that were associated with TCBV or WMH at p < 0.001 (as primary structural indicators of cellular and vascular brain damage) were also associated with at least two of the other brain MRI measures (p < 0.01). We also evaluated if genes that were associated with lower scores on either F1 or F3 at p < 0.001 (as primary indicators of amnestic, Alzheimer-type and vascular cognitive impairment) seemed associated with other cognitive test measures.

Finally, we investigated SNP associations in candidate genes. There are few candidate genes that have been directly linked in prior studies to the endophenotypes described in these analyses. Hence, we investigated genes previously reported to be associated with stroke, Alzheimer's disease, brain aging and vascular dementia in established databases including the NCBI Gene, PubMed and OMIM databases [35], the Alzforum Alzgene database http://www.alzforum.org/res/com/gen/alzgene [36], and the Science of Aging Knowledge Environment genes/intervention database http://sageke.sciencemag.org/cgi/genesdb [37]. All SNPs within 60 kb of the candidate genes (listed in Additional data file 1, Additional table 2) were examined for association with the 15 phenotypic traits described in this paper. Only phenotype-SNP associations with a p-value < 0.001 are described in Table 4.

**Table 2:** Structural and Functional Brain Aging (MRI and Cognitive Testing) Phenotypes† for FHS 100K Project: Results of Association and Linkage Analyses

2, section a: GEE, Top 25 p-values						
**Phenotype**	**SNP**	**Chromosome**	**Physical location**	**GEE p-value**	**FBAT p-value**	**Gene Region (within 60 kb)**

ATCBV	rs1970546	20	59287333	4.0 × 10^-8^	0.005	***CDH4***
F3	rs2179965*	1	88514033	1.1 × 10^-6^	0.013	
Nam	rs1155865	4	67562623	1.6 × 10^-6^	0.554	
F3	rs2832077	21	29062892	1.8 × 10^-6^	0.007	
F2	rs2352904	14	48442551	2.1 × 10^-6^	0.012	
F2	rs6914079*	6	14704344	2.2 × 10^-6^	0.018	
F2	rs9325032	5	146395409	2.8 × 10^-6^	0.008	
ALLV	rs2847476	11	113696226	3.0 × 10^-6^	0.001	*NNMT*
Sim	rs3891355	12	105453162	3.2 × 10^-6^	0.089	*POLR3B*, ***RFX4***
ATBV	rs5028798	11	34562011	3.3 × 10^-6^	0.394	*EHF*
Nam	rs530965	11	78742749	3.5 × 10^-6^	0.119	
Nam	rs9303401	17	54202944	4.9 × 10^-6^	0.099	*PPM1E*
AFBV	rs952700	11	99090946	5.7 × 10^-6^	0.003	*CNTN5*
F3	rs1031381	11	133593892	6.0 × 10^-6^	0.075	*NCAPD3*
F2	rs10489896*	1	230890353	6.2 × 10^-6^	0.109	*TARBP1*
WRAT	rs9300212	12	33592433	8.2 × 10^-6^	0.002	
Nam	rs1831521	9	90488911	8.4 × 10^-6^	0.002	*DIRAS2*
F3	rs934299	2	137172672	9.0 × 10^-6^	0.318	
ALTHBV	rs360929	4	153265305	9.1 × 10^-6^	0.055	
F2	rs2893363	7	29952294	9.6 × 10^-6^	0.812	*C7orf41*
WRAT	rs10502991	18	50243287	1.0 × 10^-5^	0.001	
APBV	rs2769965	9	79048598	1.1 × 10^-5^	0.012	
APBV	rs719435	7	31324796	1.1 × 10^-5^	0.188	*CCDC129*
F1	rs9292769	5	40433668	1.1 × 10^-5^	0.163	
Nam	rs10506718	12	75377929	1.1 × 10^-5^	0.402	

**2, section b: FBAT, Top 25 p-values**						

**Trait**	**SNP**	**Chromosome**	**Physical location**	**GEE p-value**	**FBAT p-value**	**Gene Region(s) (within 60 kb)**

ALLV	rs7124781	11	42513374	0.008	2.0 × 10^-7^	
Sim	rs1131497	11	121007955	0.008	3.2 × 10^-6^	***SORL1***
WRAT	rs10506065	12	30342307	0.050	5.0 × 10^-6^	
AFBV	rs3852286	7	140126618	0.145	6.5 × 10^-6^	*BRAF and MRPS33*
WRAT	rs4529807	10	22358107	0.013	1.1 × 10^-5^	*DNAJC1*
F3	rs847342	14	71805791	0.441	1.3 × 10^-5^	*RGS6*
AFBV	rs719775	3	64366493	0.001	1.8 × 10^-5^	
Sim	rs936111	15	99376659	0.014	2.1 × 10^-5^	***LRRK1***
ATBV	rs2143881	6	50960846	0.077	2.1 × 10^-5^	*TFAP2B*
AHPV	rs9293140	5	24643203	0.092	2.1 × 10^-5^	*CDH10*
AFBV	rs9288446*	2	212907533	0.001	2.3 × 10^-5^	***ERBB4***
APBV	rs1472962	4	95949555	0.004	3.1 × 10^-5^	***PDLIM5***
ATBV	rs2793772	13	99029574	0.047	3.3 × 10^-5^	*CLYBL*
F2	rs1333583	13	82037151	0.031	3.4 × 10^-5^	
ATBV	rs10497352	2	170781278	0.005	3.6 × 10^-5^	*ZNF650*
F1	rs497836	13	93605509	0.020	3.8 × 10^-5^	*GPC6*
APBV	rs6459928	7	158428045	0.271	4.0 × 10^-5^	***VIPR2***
AHPV	rs1963442	3	75872661	0.046	4.3 × 10^-5^	*ZNF717*
APBV	rs10503238	8	4027465	0.002	4.4 × 10^-5^	
F2	rs2861215	2	77958447	0.006	4.7 × 10^-5^	
Nam	rs9311168	3	37952421	0.067	4.9 × 10^-5^	*CTDSPL*
F2	rs2029395	2	1.8 × 10^-8^	0.027	4.9 × 10^-5^	*TTN*, *FLJ39502*
ATCBV	rs10510717	3	41307494	0.005	5.0 × 10^-5^	***CTNNB1***
ATBV	rs1433527	2	1.8 × 10^-8^	0.028	5.1 × 10^-5^	*DDX18*

**2, section c: Linkage Peaks with LOD scores ≥ 2.0.**						

**Trait**	**SNP closest to linkage peak**	**Chromosome**	**Physical location**	**1.5 – LOD support interval start**	**1.5 – LOD support interval end**	**LOD score**

WATBV†	rs1547275	9	79548023	76128637	86702472	2.81
WF3†	rs2975420	8	19534278	12651557	22836499	2.20
WNam†	rs2765241	1	62439617	59085658	67006164	2.95
WNam†	rs293966	11	26536069	21237681	33363547	2.14
WWRAT†	rs10512187	9	87400439	84893406	110115339	2.04
WWRAT†	rs1328822	13	93605666	87815515	97536766	2.50
**WWRAT†**	**rs1846090**	**18**	**14573728**	**13423610**	**19583575**	**5.10**
WWRAT†	rs10518241	19	3540074	1888178	6189414	2.33
**BMRIZLWMHVMV**	**rs4426714**	**4**	**5052671**	**105905**	**9505355**	**2.20**
BMRIZLWMHVMV	rs236535	17	65788911	59677087	68475624	2.09

Autosomal SNPs with genotypic call rate ≥ 80%, minor allele frequency ≥ 10%, Hardy-Weinberg test p ≥ 0.001, and ≥10 informative families for FBAT. Genes in bold are highlighted in discussion

*Indicates a similar result for this trait was observed (but not shown) for a SNP with r^2 ^= 1 to the reported SNP

^†^Winsorized residuals were analyzed, hence trait names are prefixed with a 'W'; linkage results in bold are highlighted in the discussion

**Table 4:** Phenotypic Associations With Candidate Genes Previously Related To Stroke, Dementia And Brain MRI Or Cognitive Function Phenotypes: Phenotype-SNP Associations With A GEE Or FBAT P-Value < 0.001

Gene	Phenotype	SNP	Chr	Physical Position	GEE_Pvalue	FBAT_Pvalue
***NGFB***	AHPV	rs10489531	1	115495044	9.7 × 10^-4^	0.046
*PRSS25*	ATBV	rs363685	2	74726491	2.5 × 10^-4^	
	ATCBV	rs363685	2	74726491	5.7 × 10^-5^	
*GRK4*	F1	rs2105380	4	3019934	0.276	5.8 × 10^-4^
*HMGE*	ATBV	rs4689584	4	7179291	0.035	1.0 × 10^-4^
*APBB2*	F1	rs10517001	4	41051593	1.4 × 10^-4^	0.009
*SNCA*	Sim	rs3796661	4	91044685	2.6 × 10^-4^	0.332
	AHPV	rs2870028	4	91122391	3.2 × 10^-4^	0.180
	AHPV	rs7678651	4	91125549	1.3 × 10^-4^	0.102
	AHPV	rs10516848	4	91132390	1.6 × 10^-4^	0.115
***PDE4D***	WRAT	rs295973	5	58945866	5.3 × 10^-4^	0.060
	Sim	rs10514882	5	59170282	6.4 × 10^-5^	
	Sim	rs9292216	5	59187886	2.4 × 10^-4^	
*DCDC2*	F3	rs10484657	6	24224829	0.017	9.5 × 10^-4^
	ATCBV	rs10484657	6	24224829	6.0 × 10^-4^	0.015
*THBS2*	ATBV	rs6937001	6	169382718	6.0 × 10^-4^	0.021
*GATA4*	WRAT	rs7006733	8	11645399	0.034	3.1 × 10^-4^
***NRG1***	ATCBV	rs1383893	8	31844190	0.407	8.9 × 10^-4^
	F2	rs10503906	8	32291504	0.187	4.2 × 10^-4^
	AFBV	rs10503919	8	32519284	1.9 × 10^-4^	0.288
	BMRIZLWMH	rs10503926	8	32660758	8.7 × 10^-5^	0.009
	VMV	rs10503927	8	32662772	2.0 × 10^-4^	0.002
***VLDLR***	ATCBV	rs502309	9	2562909	5.4 × 10^-6^	0.157
	Nam	rs2168136	9	2584577	1.6 × 10^-4^	0.006
***NTRK2***	F1	rs10512152	9	84457363	7.6 × 10^-5^	0.018
	AFBV	rs1573219	9	84617176	0.174	5.6 × 10^-4^
	AFBV	rs7038866	9	84617461	0.146	3.6 × 10^-4^
TEK	ATBV	rs628873	9	27162838	4.9 × 10^-4^	0.051
***BACE1***	APBV	rs1261791	11	116705848	0.021	3.1 × 10^-4^
***SORL1***	Sim	rs1131497	11	121007955	0.008	3.2 × 10^-6^
	Sim	rs726601	11	120986617	0.046	8.2 × 10^-4^
*VWF*	ATCBV	rs216903	12	5975760	0.149	9.5 × 10^-4^
***A2M***	F3	rs2889717	12	9178068	9.3 × 10^-5^	
***LRRK2***	WRAT	rs2249017	12	38847487	5.5 × 10^-4^	0.061
	F1	rs7975693	12	38874945	8.1 × 10^-5^	0.005
	AFBV	rs10506151	12	38957265	3.7 × 10^-4^	1.6 × 10^-4^
	ATBV	rs10506151	12	38957265	9.7 × 10^-4^	0.371
*CNTN1*	ATBV	rs10506176	12	39561067	8.8 × 10^-4^	0.622
***LTA4H***	Sim	rs10492226	12	94906457	6.7 × 10^-4^	
*IGF1*	ALLV	rs1980236	12	101270359	0.046	9.3 × 10^-4^
*LTB4R2*	APBV	rs724165	14	23876069	4.5 × 10^-4^	0.162
***NTRK3***	ALLV	rs10520671	15	86347520	6.2 × 10^-5^	0.062
*CCL2*	WRAT	rs1024612	17	29573469	0.231	7.0 × 10^-4^
*PRKCA*	F1	rs9303511	17	62158093	0.068	7.9 × 10^-4^
*CST3*	AOBV	rs1158167	20	23526189	6.5 × 10^-4^	0.032
***PRNP***	APBV	rs2326510	20	4649211	0.000718	0.064794
*BACE2*	F1	rs2007397	21	41438062	0.030	2.2 × 10^-4^
	F1	rs10483073	21	41499167	3.5 × 10^-4^	
	Nam	rs10483073	21	41499167	1.7 × 10^-4^	

*Genes in bold are highlighted in discussion section.

## Results

The brain aging phenotypic traits available in the Framingham Study 100K SNP resource with details of the sample size, statistical transformation and details of the covariates used for multivariable adjustment of each phenotype are provided in Table 1. The mean age of the 705 subjects was 62 ± 12 years, 46% were male, 79 were from the Original Framingham cohort (enrolled in 1948–50) while 626 belonged to the Offspring cohort. Table 2 (sections a and b) provide the top twenty-five phenotype-SNP associations ranked in order by lowest p-value for the GEE and FBAT models and Table 2 (section c) presents the phenotype-SNP associations with LOD scores ≥ 2.0 and the corresponding 1.5 – LOD support interval. The strongest phenotype-SNP association in GEE analyses was between a SNP on the retinal cadherin gene *CDH4 *and TCBV (rs1970546; p = 3.7 × 10^-8^) and this was the only association that achieved genome-wide significance if we applied a conservative Bonferroni correction as detailed in the Overview (p < 5 × 10^-8^); in FBAT analyses the strongest phenotype-gene association was between a SNP on the gene *SORL1 *(rs1131497; p = 3.2 × 10^-6^) and performance in Sim, a test of abstract reasoning. Assuming an additive genetic model, a minor allele frequency of10% and a very conservative α of 1 × 10^-8 ^we had an 80% power to detect an effect of 0.52 standard deviations (SD) in a given variable. For TCBV this translates to an effect size of 1.71% equivalent to8.5 years of brain aging.

We had previously reported high heritability for WMH. In the current analyses examining associations between individual SNPs and WMH there was one association that was in the top 50 and others that were in the top 100, but none were within the arbitrarily chosen cut-off for Table 2 which only details the top 25 phenotype-SNP associations. In FBAT analyses, rs1822285 and rs166085, on chromosomes 11 and 5 respectively, were associated with WMH (p = 6.4 × 10^-5 ^and 9.3 × 10^-5^) but these SNPs are not within known genes. In GEE analyses, two SNPs on the biologically plausible gene *CLDN10 *or claudin 10, an integral membrane protein that is a component of the tight junction, were related to WMH (rs10508012 and rs10508013, p = 3.3 × 10^-5 ^and 4.9 × 10^-5^). Other extragenic SNPs and SNPs on biologically interesting genes (the glial growth factor *NRG1 *and the potassium channel protein *KCNMA1*) were also associated with WMH with p values in the 10^-5 ^to 10^-4 ^range. We again observed the linkage between WMH and a region on chromosome 4 that we had previously reported [38]. Within this linkage peak (1.5 LOD support interval) were biologically interesting candidate genes such as *EVC *and *EVC1 *related to the Ellis van Creveld syndrome and *GRK4*, previously related to salt-sensitive hypertension [39,40].

We observed that performance on the Wide-Range Achievement Test (WRAT), a test of reading ability, was linked to a region on chromosome 18p with a maximum LOD score of 5.1 at rs1846090. The 1.5 LOD support interval of this linkage peak includes an STR marker, D18S53, that has been associated with dyslexia in some prior studies [41] although not in others [42]. In the current study the observed LOD score for WRAT at D18S53 (GATA11A06) was 2.5.

Table 3 provides all phenotype-SNP associations with a GEE or FBAT p < 0.001 for a key phenotype identified a priori, and a GEE or FBAT p < 0.01 for at least two other phenotypes within each of two groups of related phenotypes. These two groups were the brain MRI parameters (with TCBV and WMH as the key phenotypes) and the cognitive tests (run once with F1 and once with F3 as the key phenotype). If adjacent SNPs were in significant linkage disequilibrium [LD] (r^2 ^> 0.80) results are only presented for the strongest phenotype-SNP association noted within the LD block. For the MRI parameters, GEE models identified 10 SNPs and FBAT models identified 7 SNPs using TCBV as the index phenotype and none using WMH as the index phenotype; among these were 4 SNPs on *PDE3A *and one each on *PDE4B *and *SCN8*. For the cognitive phenotypes GEE models identified 7 phenotype-SNP associations using F1 as the key phenotype and 4 using F3 as the key phenotype; FBAT models did not identify any phenotype-SNP associations meeting these prespecified criteria.

**Table 3:** SNP Associations with a GEE or FBAT p-value < 0.001 for selected phenotype and p values < 0.01 for at least two other phenotypes within selected group of related phenotypes

Selected Phenotype : (p < 0.001)	SNP	Chr	Physical Position	Gene	MRI phenotype showing strongest association with SNP	GEE p-value
**TCBV**	rs646860	1	60310322	*C1orf87*	APBV	6.5 × 10^-4^
**TCBV**	rs7763081	6	53816274	*LRRC1*	ATCBV	6.5 × 10^-4^
**TCBV**	rs1444644	12	20457227	***PDE3A***	ATBV	8.8 × 10^-5^
**TCBV**	rs10505865	12	20453861	***PDE3A***	AFBV	1.4 × 10^-4^
**TCBV**	rs1444645	12	20457264	***PDE3A***	ATBV	1.8 × 10^-4^
**TCBV**	rs1444629	12	20454174	***PDE3A***	ATCBV	2.7 × 10^-4^
**TCBV**	rs303816	12	50469752	***SCN8A***	ATCBV	6.1 × 10^-4^
**TCBV**	rs2827980†	21	23524857		ATCBV	5.9 × 10^-5^
**TCBV**	rs9297594†	8	120287483		AFBV	1.3 × 10^-4^
**TCBV**	rs10512927†	5	50346833		ATCBV	8.5 × 10^-4^

**Selected Phenotype : (p < 0.001)**	**SNP**	**Chr**	**Physical Position**	**Gene**	**Phenotype showing strongest association with SNP**	**FBAT p-value**

**TCBV**	rs7740148	6	35063681	*ANKS1*	AFBV	0.003
**TCBV**	rs6496742	15	89324040	*PRC1*	ATCBV	6.4 × 10^-4^
**TCBV**	rs2788646	1	66518974	***PDE4B***	ATCBV	3.3 × 10^-4^
**TCBV**	rs10500956†	11	23435031		AFBV	0.003
**TCBV**	rs2059943†	8	107140783		ATCBV	1.4 × 10^-4^
**TCBV**	rs853256†	3	64290504		AFBV	7.5 × 10^-4^
**TCBV**	rs853260†	3	64289592		AFBV	4.4 × 10^-4^

**Selected Phenotype : (p < 0.001)**	**SNP**	**Chr**	**Physical Position**	**Gene**	**Phenotype showing strongest association with SNP**	**GEE p-value**

**F1**	rs4733809	8	1.29E+08	*TMEM75*	Sim	1.5 × 10^-4^
**F1**	rs3923615	11	24638108	*LUZP2*	F1	1.1 × 10^-4^
**F1**	rs10515155	17	53836943	*RNF43*	F1	3.6 × 10^-^
**F1**	rs1204116	6	62462055	*KHDRBS2*	F1	7.3 × 10^-4^
**F1**	rs708891	12	1.18E+08	*CCDC60*	F1	4.7 × 10^-4^
**F1**	rs10515159	17	54157692	*RAD51C*	F1	3.1 × 10^-4^
**F1**	rs10506214	12	41397957		F3	5.8 × 10^-4^
**F3**	rs608825	1	2.33E+08	*EDARADD*	F3	5.5 × 10^-4^
**F3**	rs957603	15	38796960	*RAD51*	F3	1.5 × 10^-4^
**F3**	rs10506214†	12	41397957		F3	5.8 × 10^-4^
**F3**	rs2109479	5	56979996		F3	4.2 × 10^-4^

Table is ordered by primary phenotype (TCBV, F1 or F3; whether significant phenotype-SNP association was based on GEE or FBAT p-value and then alphabetically by gene name.

* Genes in bold and highlighted in discussion; † SNPs were not within 60 KB of a known gene.

We identified 163 potential candidate genes and looked for phenotype-SNP associations using all SNPs on the 100K Affymetrix gene chip that were within 60 kb of the candidate gene. 23 genes had no analyzable SNPs within the 100K Affymetrix gene chip while 140 genes had 1430 analyzable SNPs within 60 kb of the gene. Table 4 shows the candidate genes and all phenotype-SNP associations with a GEE or FBAT p-value < 0.001. In this analysis we included all SNPs regardless of MAF since in prior studies significant phenotype-SNP associations had been demonstrated for some of these genes with SNPs having MAF < 10%.

## Discussion

This is the first GWA study of volumetric brain MRI and cognitive phenotypes in a community-based sample of adults with data drawn from two generations of persons within the same families. The complete results of the association and linkage analyses are available at our website http://www.ncbi.nlm.nih.gov/projects/gap/cgi-bin/study.cgi?id=phs000007. This resource has the potential to detect novel susceptibility genes for brain aging, to examine the relevance within humans of promising candidate gene associations with these diseases reported in animal models, and to replicate findings observed in other cohort studies. We used several strategies to prioritize phenotype-SNP associations, but there remain other unique ways of looking at these data that we and others will continue to explore.

In our untargeted approach of ranking SNP associations by the strength of the p-value, we found several phenotype-SNP associations within biologically interesting genes (Table 2). The most exciting was a strong association between two SNPs in or adjacent to the gene *SORL1 *and performance on tests of abstract reasoning (rs1131497; FBAT p = 3.2 × 10^-6 ^and rs726601; FBAT p=8.2 X 10^-4^). *SORL1 *is an apolipoprotein E receptor, binds alpha-2-macroglobulin, and is one component of a large multimeric complex, termed the retromer complex that is involved in retrograde transport of proteins from endosomes to the trans-Golgi network [43,44]. This retromer complex appears to play a crucial role in the transportation of transmembrane proteins implicated in Alzheimer's disease, such as amyloid precursor protein (*APP*) and β-site APP cleaving enzyme (*BACE1*). SORL1 protein is underexpressed in the frontal lobes of persons with AD compared to controls and the *SORL1 *gene has recently been associated with the risk of developing AD in 6 population samples [45,46]. Only 7 SNPs on or adjacent to the *SORL1 *gene were evaluated in the 100K Affymetrix gene chip. One of these SNPs on *SORL1 *that was associated with abstract reasoning (rs726601, FBAT p = 8.2 × 10^-4^, Table 4) was in LD (r^2 ^> 0.8) with SNPs (rs2282649, rs1010159) strongly associated with AD in these studies [45,46].

In unbiased analyses, we also identified 3 genes that were associated with measures of frontal or parietal brain volume and with tests of executive function and abstract reasoning. These 3 genes, *ERBB4*, *PDLIM5 *and *RFX4*, (FBAT ranks #11 and 12, GEE rank #9) have each been previously associated with schizophrenia or mood disorders, conditions known to be associated with smaller frontal brain volumes and poorer performance on tests of executive function, even in unaffected family members [47,48]. *ERBB4 *is a neuregulin (*NRG1*) receptor involved in forebrain development and N-methyl-D-aspartate (NMDA) receptor function. It has been associated with schizophrenia wherein excess of the IVS 12–15C > T has been noted (odds-ratio 2.98) [49,50]. *NRG1 *itself has been associated with schizophrenia in the Icelandic DeCODE population [51] and in other studies [52-54], with accelerated lobar atrophy [52], and with bipolar disorders [55,56]. As shown in Table 4, *NRG1*, like *ERBB4*, was associated with frontal brain volume (FBV) in our sample. *PDLIM5 *polymorphisms have been associated with schizophrenia (rs2433320 and rs2433322) [52,55] and bipolar disorder (rs10008257 and rs2433320) [57]. Additionally the PDLIM5 protein is a homolog of AD7c-NTP, a neural thread protein associated with Alzheimer's disease, and is being studied as a possible CSF biomarker of AD [58]. A final group of 3 genes, *CDH4*, *VIPR2*, *CTNNB1 *(GEE rank #1 and FBAT ranks #17 and 24) have been shown in animal studies to play an important role in neural tract and synaptic development [59-61]. Using linkage analyses, we were able to replicate a previous report that dyslexia was linked to a short-tandem repeat marker D18S53 on chromosome 18p11.2.

We examined pleiotropic effects by identifying SNP associations across two sets of related phenotypes. In these analyses, we uncovered a different set of genes, none of which have been related to brain volumes, cognitive function, stroke or dementia in prior population studies. However, there are biologically interesting genes related to brain volumes including *PDE3A*, previously related to all aspects of thrombosis [62], *SCN8A *linked to cerebellar ataxia with mental retardation [63], and *PDE4B *which has been associated with schizophrenia [64].

We also evaluated SNPs within some candidate genes previously reported to be associated with stroke and dementia in animal studies or in population samples, and observed that several of these SNPs were associated with MRI and cognitive endophenotypes that increase the risk of these conditions; this gene list is representative but not comprehensive. Among these genes are *PDE4D *and *LTA4H *that have been previously related to stroke in several population samples [9,10]; *NGFB*, *NTRK2 *and *NTRK3 *(a neural growth factor and two receptors for neural growth factors) genes, previously associated with performance on memory tasks in animal studies [65,66]; *BACE1*, *PRNP *and *A2M*, genes associated with AD in case-control or family-based association studies [36,67,68], *VLDLR*, a gene previously associated with an increased risk of dementia in the presence of vascular risk factors [69] and *LRRK2*, a gene associated with an increased risk of Parkinson's disease in population samples [70], but also thought to be an enabling gene for tau pathology [71]. There has been only one prior study that directly related a gene (*KIBRA*) to one of the phenotypes (verbal memory) included in the current analyses. We did not have any SNPs in significant LD with the SNP (rs17070145) described in that study [72]. We have chosen not to include details of the correlation between SNPs from the 100K and the specific SNP(s) studied within candidate genes by prior investigators since doing so would have expanded our Table 4 beyond the size and scope of this article. For example, prior associations of several of the candidate genes with related clinical disease phenotypes (for example, *PDE4D *with ischemic stroke, *SORL1 *with AD) have described allelic heterogeneity. In these studies, multiple SNPs and haplotypes within the gene were associated with the phenotype, even within Caucasian populations [73-75].

## Limitations

Our study had several limitations. A healthy survivor bias is likely as participants in this sample had to survive beyond 1990 to provide DNA. Further, persons undergoing MRI had to travel to an MRI center, provide informed consent, and have no contraindication to the study. We have previously shown that persons undergoing brain MRI were significantly healthier than the overall sample of Framingham participants alive at the time [15].

Our sample of 705 *related *persons may have a limited power to uncover associations as compared to the larger sample that includes unrelated subjects (on whom 100K genotyping was not obtained). This is especially true for hippocampal volumes, which were computed based on hand-drawn hippocampal outlines; the number of persons in our study dataset with available hippocampal volumes was only 327. Further, we currently have only a single measure of brain MRI and cognitive tests in these subjects. However, all these participants are being restudied with a second cycle of MRI and cognitive testing. The genes associated with *changes *in these measures over time may be stronger candidate genes for usual and pathological brain aging processes than the genes related in current analyses to cross-sectional endophenotypes.

The 100K Affymetrix GeneChip provides limited (~30%) coverage of the genome, with no coverage of several gene rich areas and key candidate genes such as *APOE *[76]. However, the forthcoming NHLBI funded 550 K genome-wide scan on over 9000 Framingham participants (discussed in the Overview) should permit validation of our initial 100K SNP associations in a larger sample and will provide more dense coverage of the genome. Population stratification is not a major concern in this study sample due to the high homogeneity of ancestry (European). However, for the same reason we cannot detect race or ethnicity-specific variations in these phenotype-SNP associations. There are significant issues of multiple-testing which are addressed in the Overview; when testing for association with all alleles having a minor allele frequency >5%, it has been estimated that 1,000,000 tests are conducted across the entire human genome, hence for an α of 0.05, using a conservative Bonferroni correction (0.05 × 10^-6^) only tests with a p value < 5 × 10^-8^) would be considered significant; however others have argued that this is too stringent a threshold since it ignores correlation between individual SNPs [77-79]. We emphasize that the current study is hypothesis-generating and our findings need to be replicated in other population samples.

## Conclusion

The untargeted genome-wide approach to detect genetic associations with brain aging identified several biologically interesting genes (such as genes previously related to AD and schizophrenia) as possible novel candidates related to brain structure and function in middle-aged to elderly populations. Our data also suggest that genes previously associated with clinical disease may be associated with clinical endophenotypes known to increase the risk of developing these conditions. Finally, our database will serve as a resource for in silico replication of findings noted in other population-based samples, and in animal models of brain aging, stroke, and neurodegenerative diseases.

## Abbreviations

GWAS = Genome-wide association study; FBAT = family based association testing; GEE = generalized estimating equations; LOD = logarithm of the odds; SNP = single nucleotide polymorphism; MRI = Magnetic resonance imaging; AD = Alzheimer's disease; TCBV = Total Cerebral Brain Volume; WMH = White matter hyperintensity volume; CSF = Cerebrospinal fluid.

## Competing interests

The authors declare that they have no competing interests.

## Authors' contributions

SS was involved in phenotype definition and data collection, planned the analyses, drafted and critically revised the manuscript. ALD planned and conducted the analyses and assisted in writing and critically revising the manuscript. RA was primarily responsible for the definition of cognitive phenotypes, supervised the collection of cognitive and MRI phenotypes and assisted in securing funding, planning the analyses and critically revising the manuscript. JMM and ASB were involved in phenotype definition, planning and conducting the analyses and critically revising the manuscript. CSK and MKH helped define and collect stroke-related phenotypic data and critically revised the manuscript. CD supervised the generation of MRI measurements, helped plan the analyses and reviewed the manuscript. RBD contributed to phenotypic definition, planning of analyses and helped critically revise the manuscript. LDA helped plan and conduct the analyses and critically revised the manuscript. PAW conceived of the Framingham 'MRI, Genetic and Cognitive Precursors of AD and Dementia' study, obtained funding for phenotype collection, helped plan the analyses and critically revised the manuscript. All authors read and approved the final manuscript.

## Supplementary Material

Additional file 1 Details of test source and parameters used to define each individual test and factor are outlined in Additional Table 1. All SNPs within 60 kb of the candidate genes are listed in Additional Table 2. Click here for file
