# Supplementary material for: Genetic correlates of brain aging on MRI and cognitive test measures: a genome-wide association and linkage analysis in the Framingham study
Source: BMC Med Genet. 2007 Sep 19;8(Suppl 1):S15. doi: 10.1186/1471-2350-8-S1-S15 (PMC1995608; doi:10.1186/1471-2350-8-S1-S15)
Supplement: Additional file 1 — Details of test source and parameters used to define each individual test and factor are outlined in Additional Table 1. All SNPs within 60 kb of the candidate genes are listed in Additional Table 2. [file 1471-2350-8-S1-S15-S1.doc]

##### **Additional file:** Table 1: Description of Cognitive Phenotypes

| **Cognitive Factors** | **Major cognitive domains assessed** | **Measures of performance summed to yield total score** |
| --- | --- | --- |
| **FACTOR 1: Verbal Memory (VM):** | | |
| WMS^‡^ Logical memory – Paragraph A | Verbal memory | Immediate Recall (LM-IR); Delayed Recall (LM-DR); Delayed recognition (LM-Rec) |
|  |  |  |
| **FACTOR 2: Visuospatial Memory & Organization (VMO):** | | |
| WMS Visual reproductions | Visual memory | Immediate Recall (VR-IR); Delayed Recall (VR-DR); Delayed recognition (VR-Rec) |
| Hooper Visual organization^4^ | Visual perception | Total score (VOT) |
|  | | |
| **FACTOR 3: Attention and Executive Function :** | | |
| Trails A and B^§^ | Simple attention, Executive function | Time to completion (minutes) for each test (TMT-A; TMT-B) |
| **Additional Tests** | | |
| WAIS ^†^ Similarities | Abstract reasoning | Total score (Sim) |
| Boston Naming Test (30 items) | Language-naming | Total score without cues (BNT) |
| Wide Range Achievement Test - Reading | Reading, Native intelligence | Total raw score (WRAT) |

^†^ Wechsler Adult Intelligence Scale

^‡^ Wechsler Memory Scale

^§^ Halstead Reitan Neuropsychological Test Battery

***Additional file:* Table 2: Candidate Gene List for Brain Aging (MRI Volumes and Cognitive Function) Phenotypes**

| **Gene** | **Chromosome** | **Start** | **End** | **Number of SNPs in 100K Affymetrix Gene Chip within 60 Kb of gene** | **Number of SNPs used in analyses** |
| --- | --- | --- | --- | --- | --- |
| *A2M* | 12 | 9051576 | 9219754 | 6 | 6 |
| *ACE* | 17 | 58848165 | 59012935 | 0 | 0 |
| *ADCY8* | 8 | 131801728 | 132183854 | 30 | 29 |
| *ADCY9* | 16 | 3892653 | 4166187 | 6 | 6 |
| *ADD1* | 4 | 2822589 | 3028794 | 4 | 4 |
| *ADM* | 11 | 10223217 | 10345499 | 5 | 5 |
| *ADRB2* | 5 | 148125000 | 148248447 | 13 | 11 |
| *AGT* | 1 | 227085019 | 227216602 | 8 | 8 |
| *AGTR1* | 3 | 149838362 | 150003486 | 15 | 15 |
| *ALOX5* | 10 | 45129634 | 45321567 | 5 | 5 |
| *ALOX5AP* | 13 | 30147668 | 30296556 | 13 | 12 |
| *ANXA2* | 15 | 58366642 | 58537477 | 3 | 3 |
| *ANXA5* | 4 | 122886752 | 123035781 | 6 | 6 |
| *APBB2* | 4 | 40597166 | 41117416 | 19 | 19 |
| *APOA1* | 11 | 116151680 | 116273334 | 2 | 2 |
| *APOE* | 19 | 50040878 | 50164489 | 1 | 1 |
| *APP* | 21 | 26114732 | 26525003 | 23 | 21 |
| *BACE1* | 11 | 116601624 | 116752182 | 7 | 7 |
| *BACE2* | 21 | 41401597 | 41630394 | 16 | 15 |
| *BDNF* | 11 | 27573019 | 27759872 | 11 | 10 |
| *BMP6* | 6 | 7612008 | 7886726 | 13 | 13 |
| *CACNA1A* | 19 | 13119114 | 13538317 | 2 | 2 |
| *CAMK2G* | 10 | 75182264 | 75364349 | 4 | 4 |
| *CBS* | 21 | 43286369 | 43429493 | 1 | 1 |
| *CCL2* | 17 | 29546408 | 29668331 | 11 | 11 |
| *CD14* | 5 | 139931504 | 140052956 | 3 | 3 |
| *CHRNA4* | 20 | 61388514 | 61523139 | 0 | 0 |
| *CHRNB2* | 1 | 151293329 | 151422156 | 2 | 2 |
| *COL3A1* | 2 | 189604604 | 189762978 | 4 | 4 |
| *COL4A1* | 13 | 109539311 | 109817459 | 10 | 10 |
| *COMT* | 22 | 18243862 | 18391084 | 3 | 3 |
| *CPB2* | 13 | 45465322 | 45637169 | 13 | 13 |
| *CRMP1* | 4 | 5880563 | 6072857 | 2 | 2 |
| *CRP* | 1 | 156435152 | 156557452 | 8 | 6 |
| *CSF2* | 5 | 131377383 | 131499758 | 0 | 0 |
| *CST3* | 20 | 23502293 | 23626574 | 7 | 6 |
| *CTNNA3* | 10 | 67289937 | 69185933 | 134 | 128 |
| *CYBA* | 16 | 87177198 | 87304950 | 1 | 1 |
| *CYP11B2* | 8 | 143928976 | 144056261 | 0 | 0 |
| *DCDC2* | 6 | 24221713 | 24526259 | 10 | 9 |
| *DES* | 2 | 220048603 | 220176964 | 0 | 0 |
| *DRP1* | 12 | 32663523 | 121840833 | 83 | 78 |
| *ECE1* | 1 | 21230390 | 21422213 | 1 | 0 |
| *EDN1* | 6 | 12338551 | 12465070 | 2 | 2 |
| *ERG* | 21 | 38615361 | 39015488 | 31 | 27 |
| *ESR1* | 6 | 152160799 | 152576520 | 22 | 21 |
| *ESR2* | 14 | 63560702 | 63934567 | 8 | 6 |
| *F10* | 13 | 112765128 | 112911842 | 1 | 1 |
| *F13A1* | 6 | 6029316 | 6325901 | 25 | 23 |
| *F13B* | 1 | 193679977 | 193828021 | 5 | 5 |
| *F2* | 11 | 46637350 | 46777634 | 0 | 0 |
| *F2R* | 5 | 75987546 | 76127054 | 7 | 5 |
| *F2RL1* | 5 | 76090609 | 76226895 | 0 | 0 |
| *F3* | 1 | 39448280 | 94779336 | 2 | 2 |
| *F5* | 1 | 166155066 | 166347379 | 15 | 13 |
| *F7* | 13 | 112748105 | 112882346 | 1 | 1 |
| *FABP2* | 4 | 120536008 | 120660921 | 10 | 10 |
| *FGA* | 4 | 155801884 | 155929501 | 1 | 1 |
| *FGB* | 4 | 155781767 | 155909841 | 1 | 1 |
| *FGG* | 4 | 155822893 | 155951408 | 2 | 2 |
| *GAPDHS* | 19 | 40656203 | 40788058 | 2 | 2 |
| *GATA4* | 8 | 11539161 | 11714918 | 12 | 11 |
| *GNB3* | 12 | 6759635 | 6886818 | 0 | 0 |
| *GRIN2A* | 16 | 9702922 | 10243371 | 26 | 25 |
| *GRIN2B* | 12 | 13545410 | 14084319 | 38 | 35 |
| *HMGE* | 4 | 7119851 | 7247872 | 6 | 6 |
| *ICAM1* | 19 | 10182778 | 10318291 | 0 | 0 |
| *IDE* | 10 | 94143962 | 94383813 | 4 | 4 |
| *IGF1* | 12 | 101232143 | 101436808 | 7 | 6 |
| *IL10* | 1 | 203269342 | 203394234 | 4 | 4 |
| *IL1A* | 2 | 113187723 | 113319202 | 3 | 3 |
| *IL1B* | 2 | 113243567 | 113370587 | 4 | 3 |
| *IL1RN* | 2 | 113531700 | 113667823 | 8 | 6 |
| *IL6* | 7 | 22480059 | 22604856 | 6 | 6 |
| *IL6R* | 1 | 151130741 | 151313261 | 3 | 3 |
| *IL8* | 4 | 74911309 | 75034466 | 5 | 4 |
| *ITGA2* | 5 | 52261013 | 52483947 | 14 | 14 |
| *ITGA2B* | 17 | 39745075 | 39882399 | 1 | 0 |
| *ITGB3* | 17 | 42626206 | 42805076 | 5 | 5 |
| *KIBRA* | 5 | 167591669 | 167889340 | 10 | 10 |
| *LETM1* | 4 | 1721990 | 1885205 | 0 | 0 |
| *LMNA* | 1 | 152837570 | 152982944 | 1 | 1 |
| *LPL* | 8 | 19781057 | 19929049 | 8 | 8 |
| *LRP8* | 1 | 53363232 | 53565842 | 2 | 1 |
| *LRRK2* | 12 | 38845200 | 39107834 | 20 | 19 |
| *LTA* | 6 | 31588071 | 31710077 | 0 | 0 |
| *LTA4H* | 12 | 94837078 | 94991833 | 16 | 16 |
| *LTB4R2* | 14 | 23788000 | 23910798 | 3 | 3 |
| *LTC4S* | 5 | 179093591 | 179216118 | 2 | 2 |
| *MAPT* | 17 | 41267623 | 41518611 | 18 | 18 |
| *MET* | 7 | 115846409 | 116092390 | 6 | 6 |
| *MMP3* | 11 | 102151742 | 102279550 | 8 | 8 |
| *MMP9* | 20 | 44010953 | 44138606 | 3 | 2 |
| *MPO* | 17 | 53642200 | 53773295 | 1 | 0 |
| *MTHFR* | 1 | 11720944 | 11860248 | 1 | 1 |
| *NCSTN* | 1 | 157066135 | 157201814 | 2 | 2 |
| *NDUFS4* | 5 | 52832241 | 53074924 | 4 | 4 |
| *NGFB* | 1 | 115480580 | 115652899 | 9 | 9 |
| *NGFR* | 17 | 44867665 | 45007360 | 2 | 2 |
| *NOS1* | 12 | 116053698 | 120843844 | 4 | 4 |
| *NOS2A* | 17 | 23047919 | 23211682 | 1 | 1 |
| *NOS3* | 7 | 150065794 | 150209323 | 1 | 1 |
| *NOTCH3* | 19 | 15071444 | 15232792 | 0 | 0 |
| *NPPA* | 1 | 11780041 | 11902101 | 1 | 1 |
| *NRG1* | 8 | 31556809 | 32801615 | 110 | 100 |
| *NTF3* | 12 | 5413526 | 5534725 | 1 | 1 |
| *NTF5* | 19 | 54196210 | 54318937 | 0 | 0 |
| *NTRK1* | 1 | 153583743 | 153724715 | 0 | 0 |
| *NTRK2* | 9 | 84454179 | 84926963 | 28 | 28 |
| *NTRK3* | 15 | 86160991 | 86660665 | 17 | 16 |
| *PARK7* | 1 | 7896058 | 8039602 | 5 | 3 |
| *PAWR* | 12 | 78428214 | 78647258 | 4 | 4 |
| *PAXIP1L* | 7 | 154113048 | 154265400 | 4 | 4 |
| *PDE4D* | 5 | 58242467 | 59879647 | 116 | 113 |
| *PLAT* | 8 | 42091911 | 42244351 | 0 | 0 |
| *PLAU* | 10 | 75280895 | 75407260 | 1 | 1 |
| *PLG* | 6 | 161033693 | 161204749 | 9 | 9 |
| *PNMT* | 17 | 17289601 | 35140254 | 3 | 3 |
| *PON1* | 7 | 94511638 | 94658495 | 9 | 9 |
| *PRKCA* | 17 | 61669387 | 62297324 | 26 | 25 |
| *PROC* | 2 | 127832245 | 127963048 | 0 | 0 |
| *PROCR* | 20 | 33163434 | 33288826 | 2 | 2 |
| *PROS1* | 3 | 95015718 | 95235600 | 1 | 1 |
| *PROZ* | 13 | 112800968 | 112934695 | 1 | 1 |
| *PRSS25* | 2 | 74608186 | 74732098 | 4 | 3 |
| *PSEN1* | 14 | 72612931 | 72816862 | 5 | 3 |
| *PSEN2* | 1 | 223305694 | 223450532 | 4 | 4 |
| *PTGS2* | 1 | 183312625 | 183441213 | 5 | 5 |
| *RTN4* | 2 | 55050979 | 55249385 | 8 | 8 |
| *SCNN1A* | 12 | 6266275 | 6414976 | 0 | 0 |
| *SCNN1B* | 16 | 23161140 | 23360117 | 5 | 4 |
| *SCNN1G* | 16 | 23041540 | 23195701 | 0 | 0 |
| *SELL* | 1 | 166331465 | 166472385 | 14 | 13 |
| *SELP* | 1 | 166229747 | 166391065 | 15 | 13 |
| *SERPINC1* | 1 | 170544599 | 170678130 | 2 | 2 |
| *SERPINE1* | 7 | 100303886 | 100435741 | 2 | 2 |
| *SERPINE2* | 2 | 224605374 | 224789498 | 5 | 5 |
| *SNCA* | 4 | 90943882 | 91175311 | 25 | 24 |
| *SNCB* | 5 | 175919816 | 176050163 | 1 | 1 |
| *SORT1* | 1 | 109504233 | 109712605 | 2 | 2 |
| *SORL1* | 11 | 120864475 | 121036098 | 7 | 7 |
| *TEK* | 9 | 27039463 | 27280171 | 23 | 23 |
| *TF* | 3 | 134887932 | 135040333 | 6 | 6 |
| *TFAM* | 10 | 59755181 | 59885903 | 5 | 5 |
| *TFCP2* | 12 | 49714887 | 49912931 | 2 | 1 |
| *TFPI* | 2 | 188096790 | 188304671 | 5 | 5 |
| *TGFB1* | 19 | 46468490 | 46611656 | 1 | 1 |
| *TGFB2* | 1 | 214848262 | 215063365 | 10 | 10 |
| *TGFB3* | 14 | 75434194 | 75577242 | 4 | 4 |
| *TGFBR3* | 1 | 91800003 | 92143580 | 14 | 13 |
| *THBD* | 20 | 22914270 | 23038301 | 6 | 5 |
| *THBS2* | 6 | 169373507 | 169531769 | 6 | 6 |
| *THBS4* | 5 | 79307095 | 79474861 | 5 | 3 |
| *TLR4* | 9 | 117486137 | 117617607 | 7 | 7 |
| *TNF* | 6 | 31591328 | 31714091 | 0 | 0 |
| *TRPS1* | 8 | 116429899 | 116810429 | 33 | 30 |
| *UBQLN1* | 9 | 83444432 | 83612507 | 4 | 4 |
| *UCHL1* | 4 | 41039856 | 41171373 | 8 | 8 |
| *VCAM1* | 1 | 100837317 | 100976620 | 5 | 5 |
| *VEGF* | 6 | 43785923 | 43922199 | 3 | 3 |
| *VLDLR* | 9 | 2551802 | 2704485 | 12 | 12 |
| *VTN* | 17 | 23658424 | 23781844 | 0 | 0 |
| *VWF* | 12 | 5868407 | 6163946 | 6 | 6 |
